# Use of Linear Programming to Develop Cost-Minimized Nutritionally Adequate Health Promoting Food Baskets

**DOI:** 10.1371/journal.pone.0163411

**Published:** 2016-10-19

**Authors:** Alexandr Parlesak, Inge Tetens, Jørgen Dejgård Jensen, Sinne Smed, Mojca Gabrijelčič Blenkuš, Mike Rayner, Nicole Darmon, Aileen Robertson

**Affiliations:** 1 WHO Collaborating Centre for Global Nutrition and Health, Metropolitan University College, Copenhagen, Denmark; 2 National Food Institute, Technical University of Denmark, Research Group for Risk-Benefit, Søborg, Denmark; 3 Department of Food and Resource Economics, Section for Consumption, Bioethics and Governance, Copenhagen University, Copenhagen, Denmark; 4 Nacionalni inštitut za javno zdravje—NIJZ (National Institute of Public Health), Ljubljana, Slovenia; 5 Nuffield Department of Population Health, British Heart Foundation Centre on Population Approaches for Non-Communicable Disease Prevention, Oxford University, Oxford, United Kingdom; 6 The Institut National de la Recherche Agronomique 1260 INRA, the Institut National de la Santé et de la Recherche Médicale 1062 INSERM, Aix-Marseille University, Unité Mixte de Recherche (UMR) “Nutrition, Obesity and Risk of Thrombosis”, Marseille, France; Hunter College, UNITED STATES

## Abstract

**Background:**

Food-Based Dietary Guidelines (FBDGs) are developed to promote healthier eating patterns, but increasing food prices may make healthy eating less affordable. The aim of this study was to design a range of cost-minimized nutritionally adequate health-promoting food baskets (FBs) that help prevent both micronutrient inadequacy and diet-related non-communicable diseases at lowest cost.

**Methods:**

Average prices for 312 foods were collected within the Greater Copenhagen area. The cost and nutrient content of five different cost-minimized FBs for a family of four were calculated per day using linear programming. The FBs were defined using five different constraints: cultural acceptability (CA), or dietary guidelines (DG), or nutrient recommendations (N), or cultural acceptability and nutrient recommendations (CAN), or dietary guidelines and nutrient recommendations (DGN). The variety and number of foods in each of the resulting five baskets was increased through limiting the relative share of individual foods.

**Results:**

The one-day version of N contained only 12 foods at the minimum cost of DKK 27 (€ 3.6). The CA, DG, and DGN were about twice of this and the CAN cost ~DKK 81 (€ 10.8). The baskets with the greater variety of foods contained from 70 (CAN) to 134 (DGN) foods and cost between DKK 60 (€ 8.1, N) and DKK 125 (€ 16.8, DGN). Ensuring that the food baskets cover both dietary guidelines and nutrient recommendations doubled the cost while cultural acceptability (CAN) tripled it.

**Conclusion:**

Use of linear programming facilitates the generation of low-cost food baskets that are nutritionally adequate, health promoting, and culturally acceptable.

## Introduction

In OECD countries micronutrient inadequacy can co-exist with excess calorie intake [1,2]. Vulnerable groups, especially pregnant women within low socio-economic groups and their families are at high risk [3,4]. Evidence suggests that increased intake of micronutrient-dense foods with low energy density can help to prevent nutrition-related noncommunicable diseases (NCD) along with micronutrient inadequacies [5] and corresponding national food-based dietary guidelines (FBDGs) have been developed in many countries [6]. However, micronutrient-dense foods are relatively expensive [7] so people, especially those on low incomes, buy less and this increases risk of micronutrient inadequacies [8]. Even in high-income countries, economic constraints and actual lifestyles lead people to consume diets with a low micronutrient-energy ratio [9]. Both micronutrient inadequacy and excess weight gain is expected to increase along with inequalities during economic crises even in high-income countries [10].

NCDs are the primary cause of premature morbidity in Europe [11,12]. EU and WHO Member States have called for action to prevent both NCDs and micronutrient deficiency through improved dietary practices [13,14]. There are many drivers of food purchase but the most important are: taste, availability/access, habit, and cost [15]. Governments have tried to provide positive, easy-to-understand, and readily affordable dietary guidelines in order to change population eating patterns to reduce the increasing prevalence of inequality in diet-related NCDs [1,16,17]. However the introduction of national FBDGs appears not to reduce prevalence of dietary related NCDs especially in low income groups [18].

There is a need for mathematical modelling to help calculate which foods can supply the optimum nutrient recommendations for low cost, especially for income strapped households and authorities e.g. catering services within the public sector. The method of LP has been used to optimise the average daily nutrient intake, for children and adults since the nineteen-fifties [19–22]. Several non-EU governments use LP methodology to estimate how much money their national population need to cover the cost of a nutritionally adequate diet e.g. Canada [23], Australia [24], and the United States [25]. However within Europe similar methods do not appear to be used e.g. to help governments plan their social and welfare policies.

The main aim of this study was to use linear programming methodology to design a range of cost-minimized health-promoting food baskets (FBs) that could both help to prevent micronutrient inadequacies and to be culturally acceptable for a low-income family. Five one day (24hr) low-cost FBs for a family of four were defined using five different constraints: cultural acceptability (CA) [26]; dietary guidelines (DG) [27]; nutrient recommendations (N) [6]; cultural acceptability; and nutritional adequacy (CAN), or dietary guidelines and nutrient recommendations (DGN). Realistically, family households and public catering services cannot provide the same menu to consumers day after day. In addition, the lack of variety in diets is associated with poor nutritional adequacy [28] and poor health status [29]. Hence, this study also aims to investigate how food variety within the FBs affects cost and micronutrient content and which micronutrient recommendations influence the overall cost of a healthy diet the most.

## Materials and Methods

### Generation of the list of foods usually available in Greater Copenhagen

A list of 312 unprocessed or minimally processed foods was generated. These were grouped into categories similar to 13 out of 14 food categories used in the Danish food consumption survey (Table 1) [26]. Ready meals and beverages were not included. Particular care was taken to include foods rich in vitamin D (i.e. cod liver, cod liver oil, and cod roe) due to challenges to meet vitamin D recommendations [30].

**Table 1:** Food groups and subgroups of foods with examples of foods included in the analysis.

Food group [26]	Subgroups with examples	Number of food items
Milk and milk products	Milk with varying fat content, cream, sour-fermented milk products (yoghurt, kefir etc.)	16
Cheese and cheese products	Hard and soft cheese, cottage cheese, curd	15
Cereals and other starchy foods	Bread, flour, pasta, rice, oats, bulgur, quinoa, muesli	35
Potatoes	Potatoes fresh and frozen (wedges, chips), potato products (potato flour, instant potato flakes	8
Vegetables	Leafy vegetables (cabbage, leeks, lettuce, spinach etc.)	15
	Non-leafy vegetables (tomatoes, green pepper, cucumber, broccoli, cauliflower etc.), snap beans, ketchup	21
	Root vegetables (onions, carrots, beetroot, Jerusalem artichoke, parsnip, celeriac etc.)	13
	Pulses including lentils, peas, beans, and chickpeas	16
	Mushrooms	5
Fruits, nuts and seeds	Fruits (including dried fruits)	41
	Nuts and seeds, olives	21
Juices	Apple juice, orange juice, etc.	3
Meat and meat products	Unprocessed (pork, beef, lamb) and moderately processed meat (sausage, salami etc.)	27
	Offal (liver, heart, kidneys) from pork, beef, and veal	8
Poultry	Chicken, turkey, goose, duck, chicken liver/heart and products	16
Fish and fish products	Seawater (cod, plaice, tuna, salmon) and freshwater (trout) fish, cod liver, and cod roe	21
Eggs	Eggs	1
Fats and oils	Plant oils (rapeseed, sunflower, olive), butter, margarine, coconut fat, cod liver oil, mayonnaise	14
Sugar, honey, and sweets	Sugar, honey, chocolate, chocolate bars and spread, syrup etc.	13
Condiments	Salt, vinegar	3

### Food prices

The collection of food data, including prices, was carried out within the Greater Copenhagen area (S1 Table). The price of each food was collected in five discount retailers (Netto®, Rema 1000®, Aldi®, Lidl®, and Fakta®) along with two online retailers (Nemlig.com and Superbest.com) between March and December 2014. All shop managers gave their informed consent to the collection of food prices. The lowest price was selected if one food cost a range of different prices at time of data collection. The price per kilogram of edible food was calculated in Danish Kroner (DKK) per kilogram (kg). To correct for different nutrient composition between cooked and unprepared foods, two factors were adjusted: the change in water content during preparation (before–after preparation); and the loss of pecuniary value due to cost of non-edible parts (waste such as shells, peel, skins, fruit stones, bones etc.) [31,32]. To adjust for these two factors, the following formula (1) was applied:

$${\text{Price}}_{\text{Edible food}}=\frac{{\text{Price}}_{\text{Raw food}}*({100\%\text{-}\%\text{Water}}_{\text{Content in prepared food}})*100\%}{\left({100\%\text{-}\%\text{Water}}_{\text{Content in raw food}}\right)*\%\;\text{Edible portion}}$$ (1)

If food was sold per item, 4–6 items were weighed on-site and the average weight (kg) was used to calculate the price. For the calculation of price per kg from online shops, standard weights for fruits and vegetables were used [33].

### Food Composition Tables

The Danish (Foodcomp) [31] or, where necessary, the American (SR28) [32] food composition tables and databases were used to obtain food composition values. Where appropriate, the values for prepared (cooked, baked, simmered, etc.) foods were used. Values for the average edible weight of each food were obtained from the same food composition tables and databases [31,32].

### Linear programming

Linear programming (LP) is an algorithm for maximising or minimising a given (linear) objective function subject to a set of linear constraints [34] on a list of decision variables. The decision variables were whether a food was selected and at what weight. The objective function minimized the total cost of the FB (the sum of the cost of each food in the basket). Each food was characterised by its price and its nutrient content. LP was used to design five different FBs, which were defined using the following sets of constraints (Table 2):

1. Culturally acceptable FB (CA) follows current eating patterns in Denmark [26];
2. Health-promoting FB (DG)—follows the Danish food-based dietary guidelines [27];
3. Nutritionally adequate FB (N)—meets all recommended Nordic nutrient intake values [6];
4. Culturally acceptable, nutritionally adequate FB (CAN)–both culturally acceptable and nutritionally adequate i.e. combines (i) CA and (iii) N;
5. Both health-promoting and nutritionally adequate FB (DGN)–i.e. combines (ii) DG and (iii) N;

In all cases, the goal was to minimize the cost of the FBs. The LP algorithm used is available as an MS Excel® open-source add-in (OpenSolver) [35], where the “COIN Branch and Cut” option was used to ensure the implementation of all constraints for each of the five different FBs. The nutrient recommendations [6] were applied individually to each member of a family of four: woman, aged 31–50 years; man, aged 31–50 years, one girl aged 4 years, and one boy aged 8 years (Table 2). This family combination was selected as it represents the household most commonly found in Denmark [36]. A daily FB was calculated for each family member and then merged to give a household´s FB for one day. All five different FBs provided the average daily age- and gender-adjusted energy intake (per individual) as recommended by the Nordic recommendations [6] (Table 2). All LP constraints for each FB are listed in Table 2.

**Table 2:** Constraints applied to the five food baskets (FBs): (i) culturally acceptable FB (CA) i.e. follows current consumption of 13 food categories in Denmark [26]; (ii) nutritionally adequate FB (N) i.e. meets all nutrient recommendations [6]; (iii) health promoting FB (DG) i.e. follows national food-based dietary guidelines [27], (iv) both nutritionally adequate and health-promoting FB (DGN) i.e. combines (ii) and (iii), and both nutritionally adequate and culturally acceptable (CAN) i.e. combines (i) and (ii). When ranges are given, both the upper and lower limits were applied as LP constraints. EI: energy intake; app.: applied; AC: average consumption.

	Girl	Boy	Female	Male	CA	DG	N	CAN	DGN
Age (y)	4	8	31–60	31–60					
Energy Kcal/day (MJ/day)	1403 (5.87)	1738 (7.27)	2103 (8.80)	2629 (11.0)	app.	app.	app.	app.	app.
AC of milk(products) (g/day)	398	457	273	337	app.	-	-	app.	-
AC of cheese (g/day)	21	20	41	47	app.	-	-	app.	-
AC of bread + cereals (g/day)	204	228	189	249	app.	-	-	app.	-
AC of potato(products) (g/day)	38	42	65	118	app.	-	-	app.	-
AC of vegetable + pulses (g/day)	157	158	206	191	app.	-	-	app.	-
AC of fruit(products) (g/day)	183	192	212	166	app.	-	-	app.	-
AC of juice (mL)	60	57	54	59	app.	-	-	app.	-
AC of meat + offal (g/day)	82	91	99	172	app.	-	-	app.	-
AC of poultry (g/day)	14	18	24	29	app.	-	-	app.	-
AC of fish(products) (g/day)	15	17	34	40	app.	-	-	app.	-
AC of eggs (g/day)	17	19	23	26	app.	-	-	app.	-
AC of fats + oils (g/day)	35	39	35	47	app.	-	-	app.	-
AC of sweets + chocolate (g/day)	33	36	35	38	app.	-	-	app.	-
Milk(products)	Milk with <0.7% fat only, fermented milk products <1.5% fat, daily consumption 250–500 mL; cheese with <17% fat only				-	app.	-	-	app.
Starchy foods	>75 g (children: 50/62 g) of whole grain products, >250/300 g (children: 167/207 g) of starchy foods				-	app.	-	-	app.
Ratio of food categories	Ratios of sum amounts of meat, poultry, fish, eggs, cheese: vegetable + fruit: potatoes + whole grain products = 1:2:2				-	app.	-	-	app.
Vegetables + fruits	>600 g (children: 200/248 g) vegetable + fruits, min. ½ of which is vegetable and max. 100 mL juice				-	app.	-	-	app.
Meat	No meat with >10% fat; red meat <500 g/week (children: 336/413 g/week)				-	app.	-	-	app.
Fish	>350 g total (children: 231/287 g);,>200 g fatty fish (children: 133/168 g)				-	app.	-	-	app.
Animal fats	Not allowed				-	app.	-	-	app.
Sugar + sweets	To be minimized				-	app.	-	-	app.
Proteins (% of total EI)	10–20				-	-	app.	app.	app.
Lipids (% of total EI)	25–40				-	-	app.	app.	app.
SFA (% of total EI)	<10				-	-	app.	app.	app.
MUFA (% of total EI)	10–20				-	-	app.	app.	app.
PUFA (% of total EI)	5–10				-	-	app.	app.	app.
w-3 FA (% of total EI)	>1				-	-	app.	app.	app.
Trans-FA (% of total EI)	<1				-	-	app.	app.	app.
Carbohydrates (% of total EI)	45–60				-	-	app.	app.	app.
Sugar (% of total EI)	<10				-	-	app.	app.	app.
Fibre (g/MJ)	>2	>2.25	>3	>3	-	-	app.	app.	app.
Sodium (mg)	<1440	<1920	<2400	<2400	-	-	app.	app.	app.
Potassium (mg)	>1800	>2000	>3100	>3500	-	-	app.	app.	app.
Calcium (mg)	600–2500	700–2500	800–2500	800–2500	-	-	app.	app.	app.
Magnesium (mg)	>120	>200	>280	>350	-	-	app.	app.	app.
Iron (mg)	8–25	9–25	15–25	9–25	-	-	app.	app.	app.
Zinc (mg)	6–25	7–25	7–25	9–25	-	-	app.	app.	app.
Copper (mg)	0.4–5	0.5–5	0.9–5	0.9–5	-	-	app.	app.	app.
Selenium (μg)	25–300	30–300	50–300	60–300	-	-	app.	app.	app.
Phosphorus (mg)	470–3000	540–3000	600–3000	600–3000	-	-	app.	app.	app.
Iodine (μg)	90–600	120–600	150–600	150–600	-	-	app.	app.	app.
Vit A (RAE)	>350	>400	>700	>900	-	-	app.	app.	app.
Thiamin (mg)	>0.6	>0.9	>1.1	>1.3	-	-	app.	app.	app.
Riboflavin (mg)	>0.7	>1.1	>1.2	>1.5	-	-	app.	app.	app.
Vit B6 (mg)	0.7–25	1–25	1.2–25	1.5–25	-	-	app.	app.	app.
Vit B12 (μg)	>0.8	>1.3	>2	>2	-	-	app.	app.	app.
Vit C (mg)	30–1000	40–1000	75–1000	75–1000	-	-	app.	app.	app.
Vit D (μg)	10–100	10–100	10–100	10–100	-	-	app.	app.	app.
Vit E (mg)	5–300	6–300	8–300	10–300	-	-	app.	app.	app.
Folate (μg)	80–1000	130–1000	300–1000	300–1000	-	-	app.	app.	app.
Niacin (mg)	9–900	12–900	14–900	18–900	-	-	app.	app.	app.

#### (i) Culturally acceptable food basket (CA) constraints enforced using Linear Programming (LP)

A food basket was considered the more culturally acceptable the less it deviated from the eating pattern of the Danish population [8]. The 312 foods, with Danish prices collected, were grouped into the same categories as those used in Danish food intake survey 2011–2013 [26]. The maximum relative deviation (MRD) for each food category was calculated as the difference between the total weight of food in basket minus the average weight consumed and divided by the average weight consumed within the same category [Formula (2)].

$$MRD=\frac{abs[\sum_{i}^{n}m_{i}-m_{j}(av)]}{m_{j}(av)}$$ (2)

In formula (2), the following abbreviations were used: n: number of foods in j-th food category; m_j_(av): average weight of foods consumed in j-th category. In food basket CA, the total weight of foods in each category was matched to correspond to the average age- and sex-specific amount consumed by the Danish population (MRD = 0) [26]. The categories and the corresponding values for m_j_(av) of each family member are listed in Table 2. In this table, the combination of constraints for each FB is indicated in one of the five columns to the right.

#### (ii) Health-promoting food basket (DG) and constraints enforced using LP

Food basket DG was calculated using LP to enforce the Danish food-based dietary guidelines (FBDGs) along with the appropriate ratios of food categories as recommended by the Ministry of Food, Agriculture and Fisheries of Denmark [27]. The constraints from the guidelines are listed in Table 2. For the children in the family, the absolute amounts of fish, fruits and vegetables, whole-grain products and meat were adapted proportionally to their individual energy recommendations (Table 2).

#### (iii) Nutritionally adequate food basket (N) and constraints enforced using LP

Food basket N was calculated using LP to enforce the Nordic recommended nutrient intake values [6] as constraints. The recommended values for macronutrients, fibre, and minerals/micronutrients (sodium, potassium, calcium, magnesium, iron, zinc, selenium, iodine, phosphorus, thiamine, riboflavin, niacin, folate, and the vitamins C, B6, A, E, D, and B12) [6] were calculated according to each individual´s energy recommendation (Table 2).

#### (iv) Nutritionally adequate and health-promoting food basket´s (DGN) constraints enforced using LP

The DGN food basket was calculated using LP to enforce a combination of both (ii) DG and (iii) N so that the DGN is both nutritionally adequate [6] and follows the Danish food-based dietary guidelines. For details, see Table 2.

#### (v) Nutritionally adequate and culturally acceptable food basket´s (CAN) constraints enforced using LP

The CAN food basket was calculated using LP to enforce a combination of both (i) CA and (iii) N so that the CAN is both culturally acceptable [26] and nutritionally adequate [6]. For details, see Table 2.

### Computation of shadow prices for single micronutrients in N

In LP models, constraints that are influencing the lowest cost (the objective function) are called “active constraints”. These consider the constraint that micronutrient levels must be equal to 100% of their recommended intake value. The shadow cost of a nutrient is calculated by the difference in the objective function value (the lowest cost) with and without an active constraint [37]. So that for each active constraint, its shadow cost was estimated by calculating the difference in cost between the FB with, and without, that constraint. After the nutrients with high shadow cost were identified, the foods rich in those nutrients were tested to examine how overall cost is influenced by their inclusion.

### Increased diversity (using a greater number and variety of foods) was modelled in all FBs through a step-wise reduction of the relative amount of foods within each category

Food baskets based on one day´s recommendations consist of a small number (6–12) of foods [19]. Such a restricted number of foods would be monotonous and unrealistic on a regular daily basis. Therefore in order to increase variation, the proportion of a food within a category, was limited using a systematic process where: 200%, 150%, 100%, 70%, 50%, 40%, 30%, 20%, and 10% of the average weight [m_j_(av)] according to the Danish dietary intake survey was calculated. For example: for an adult female the average consumption of milk and milk products was 273 g per day (Table 2); therefore in order to increase the number of foods within “milk products”, the proportion of the food was reduced in a step-wise process from 546 g (200%) to 27.3 g (10%). The FB was systematically re-calculated according to the minimum cost. The resulting cost, of approx. 100 foods, was calculated for 30.5 days or equivalent to a one-month ´s family food basket.

The deviation from the usual Danish eating pattern was calculated as the average relative deviation (ARD) from average food consumption [26] (Formula (3)):

$$ARD=\frac{1}{13}\sum_{j=1}^{13}\left\{\frac{abs[\sum_{i=1}^{n}m_{ij}-m_{j}(av)]}{m_{j}(av)}\right\}$$ (3)

In Formula (3), m_ij_ indicates the i-th food in the j-th food category. All other abbreviations are same as in Formula (2).

## Results

The least expensive food baskets, containing from 6 (DG) to 33 (CAN) foods, cost from ~DKK 24 (€ 3.2, N) to ~DKK 80 (€ 10.8, CAN) (Table 3). Combination of both nutrient and dietary recommendations (DGN) more than doubled the cost compared to N and making N culturally acceptable (CAN) more than tripled its cost (Table 3).

**Table 3:** Simplest and most affordable one-day food baskets (CA, DG, N, DGN, CAN) that follow the constraints listed in Table 2.

	CA		DG		N		DGN		CAN	
Food item	Weight (g)	Cost (DKK)	Weight (g)	Cost (DKK)	Weight (g)	Cost (DKK)	Weight (g)	Cost (DKK)	Weight (g)	Cost (DKK)
Milk, skimmed									101	0.50
Milk, 0.5%	1465	7.25	873	4.32	303	1.50	873	4.32		
Milk, 3.5%									1364	8.11
Cheddar, 33%	100	3.28								
Curd, 1%	29	0.87							129	3.86
Soy drink, 2.2%							620	11.75		
Rice, parboiled	646	1.80			361	1.01			227	0.63
Rice, polished									181	0.58
Wheat flour	224	1.10			102	0.50			34	0.17
Wheat kernels			1430	5.21			968	3.52	38	0.14
Rye flour			666	3.98	583	3.49	593	3.54	244	1.46
Rye flour, whole grain					275	2.29				
Bread for toasting, white					236	2.56	93	1.00	59	0.63
Oats									87	0.70
Instant potato flakes	263	1.26					535	2.57	263	1.26
Kidney beans	555	1.73	2096	6.54	1025	3.20	1390	4.34	712	2.22
Onions	157	0.43					705	1.91		
White cabbage					548	2.74				
Apples	753	6.69								
Watermelon									347	4.16
Cantaloupes									242	2.28
Limes									159	5.88
Olives									5	0.12
Juice, apple	230	1.52								
Juice, orange									230	1.68
Eggs	85	2.48							85	2.48
Medister (sausage)	444	14.80							233	7.78
Ham, pork, cured									186	9.24
Kidneys, pork			873	13.71	66	1.03	253	3.97		
Beef, minced, 15%									23	1.04
Salami									1	0.03
Chicken, whole	85	3.30							53	2.06
Chicken, breast or cut									32	1.67
Herring filets	106	7.20	175	11.87			112	7.62	6	0.43
Cod liver, canned							16	2.20	1	0.19
Salmon							47	3.38	98	7.07
Sunflower oil	151	1.69			26	0.29	41	0.46		
Margarine 70% fat	5	0.05							5	0.05
Rapeseed oil					175	2.02	91	1.06	133	1.54
Mayonnaise									11	0.23
Cod liver oil					16	3.29	4	0.87	8	1.58
Sugar	142	1.41							38	0.38
Toffee									63	6.83
Sweet drops									34	2.47
Chocolate, dark									7	0.90
Salt, iodised					20	0.05	2	0.01	3	0.01
Sums:	5440	56.87	6113	45.63	3735	23.98	6344	52.53	5443	80.34

The RIs of vitamins D, C, calcium, iodine, potassium, and riboflavin were active constraints and controlled the total cost of basket N. These micronutrients accounted for shadow prices of 10%, 9%, 5%, 3%, 3%, and 2%, respectively. Achieving the lowest cost depended on the availability of a small number of foods. The removal of specific foods that are rich in cost-controlling nutrients such as vitamin D, e.g. cod liver, cod liver oil, and cod roe, more than doubled the cost of FB N (~DKK 41, € 5.5). Similarly the removal of iodised salt resulted in an additional cost of DKK 2.7 (€ 0.4).

**Fig 1:**
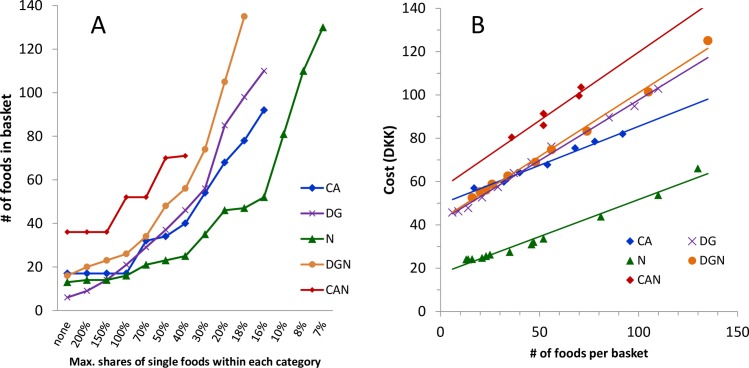
Changes of the numbers of food items in the different food baskets and their cost when subjected to diversification. A) Changes in the number of different foods in the FBs when lowering the maximum allowed relative share of any single food, expressed as percentage of the average consumption of foods in the corresponding food category. B) Change in price of the four food baskets when increasing the diversity through minimizing single food shares. All abbreviations as explained in legend of Table 2; “none” means no restriction.

An increased number (variety) of foods (Fig 1A) was created by limiting the maximum proportion of each food in all baskets. This resulted in the inclusion of 130 and 135 foods, respectively, in the N and DGN baskets compared with only 70 foods in the CAN. Attempts to increase the number of foods beyond these numbers meant that the applied constraints could no longer be met.

The cost increase was approximately linear (Pearson’s r: 0.936 (CAN) to 0.995 (DG)): it was low for N and CA (DKK 0.34 and 0.35 per additional food item, respectively) and higher for DG, DGN, and CAN (DKK 0.56, 0.59, and 0.63 per food, respectively) (Fig 1B). Out of the basic list of 312 foods, only 23 foods became part of all FBs with extended food variety and 114 were never selected to become part of any FB. Food baskets with an extended food variety contained a large number of foods and could be converted into one-month (30.5 days) baskets for a household (Table 4).

**Table 4:** Weight and price of 102 foods in an extensively diversified N food basket (max. share of a single food in each corresponding category: 8%) sufficient to provide 30.5 diversified one-day (= one month) food baskets for a family of four, costing DKK 54 (~€ 7.2) per day.

	Food item	Weight (g)	Cost (DKK)			Weight (g)	Cost (DKK)			Weight (g)	Cost (DKK)
Bread & cereals	Rice, parboiled	2123	6	Vegetables	Onions	1737	5	Milk + milk products	Milk, skimmed	3575	18
	Rice, polished	2123	7		Kidney beans	1737	5		Milk, 0.5%	3575	18
	Pasta	2123	7		Carrots	1737	8		Milk, 1.5%	3575	20
	Wheat kernels	2123	8		White cabbage	1737	9		Milk, 3.5%	3575	21
	Wheat flour	2123	10		Green lentils	1737	12		Yogurt 1.5%	2888	29
	Rye flour	2123	13		Soy beans, peeled	1737	13		Crème fraiche 38%	2825	56
	Rice, whole grain	2123	13		Red cabbage	1737	15		Buttermilk, 0.5%	1637	11
	Noodles (pasta with egg)	2123	15		White beans, small	1737	17		Yogurt 3.5%	666	8
	Barley flour	2123	15		Chickpeas	1737	18		Soured milk 3.5%	666	9
	Oats	2123	17		Tomato Ketchup	1737	18		Greek yogurt 10%	232	4
	Rye flour, whole grain	2123	18		Spinach	1737	19	Meat	Kidneys, pork	1083	17
	Wheat flour, whole grain	2123	21		Cauliflower	1354	10		Medister (sausage)	541	18
	Whole grain rye bread	2123	22		Avocado	1354	23		Kidneys, veal	200	6
	Toast, whole grain	2123	23		Celeriac	1227	11		Salami	200	7
	Baguette	2123	23		Kidney beans, canned	969	12		Heart, pork	200	10
	Bread for toasting, white	2123	23		Black beans, turtle	969	19	Fish	Cod liver, canned	259	37
	Pita bread	2123	32		Tomatoes, dried	969	51		Mackerel, filet	118	12
	Corn starch	1987	33		Red lentils	581	10		Salmon	78	6
	Couscous	1625	11		Soy beans, in husk	111	5		Trout, whole	78	10
	Pasta, whole grain	1625	12		Parsley	43	1		Cod roe	78	8
	Ciabatta	1594	32	Fruits	Oranges	1837	18		Herring filets	37	2
	Bulgur	1069	8		Cantaloupes	1144	11		Mackerel, pulled	37	4
	Spelt flour, whole grain	1069	12		Raisins	1080	34		Tuna, fresh	37	5
	Pearl barley	1069	15		Fruit jam	1019	17		Sardines in vegetable oil	37	5
	Tortillas	1069	24		Dates, dried	405	15		Plaice	37	5
	Bread, wholemeal	1069	27		Kiwis	327	6		Trout filet, smoked	37	7
	Cornmeal	1069	35		Prunes	47	2	Fats & oils	Margarine 70% fat	381	4
	Muesli (Fruit and Nuts)	1069	43		Marmalade	12	0.3		Sunflower oil	381	4
Potatoes & potato products	Instant potato flakes	642	3	Juice	Orange juice	561	4		Rapeseed oil	381	4
	Potatoes	642	4	Nuts & seeds	Sunflower kernels	1837	41		Mayonnaise	381	8
	Potato flour	642	6		Coconuts	1391	19		Cod liver oil	381	79
	Frozen chips	642	8		Sesame seeds	976	51		Corn oil	362	8
	Frozen roast potatoes	642	12		Peanuts, oil-roasted	922	44		Butter, with salt	351	13
	Potato crisps	642	17		Coconut meat, dried	222	7		Olive oil	295	11
	Frozen potato wedges	195	3		Walnuts, wo/ shell	75	9		Grapeseed oil	61	2
Sugar	Sugar	346	3	Eggs	Eggs	88	3		Duck fat	3	0.5
Salt	Salt, iodised	221	1	Cheese	Cheddar, 33%	200	7				

**Fig 2:**
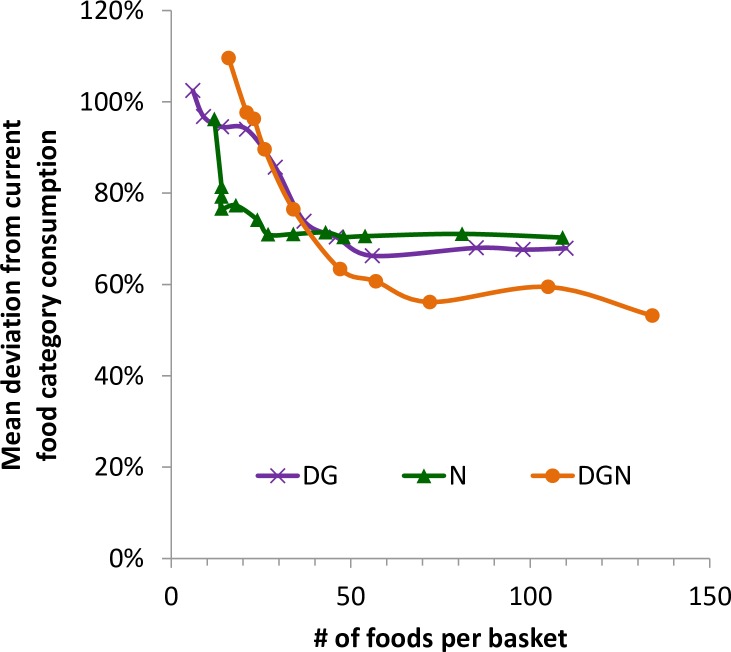
Change of the deviation of DG, N, and DGN from the average relative consumption (ARD) of food categories in Denmark when subjected to diversification.

The average relative deviation (ARD) from the usual Danish eating pattern decreased to around two-thirds (60–70%) after 50 foods were added to the basket (Fig 2).

**Fig 3:**
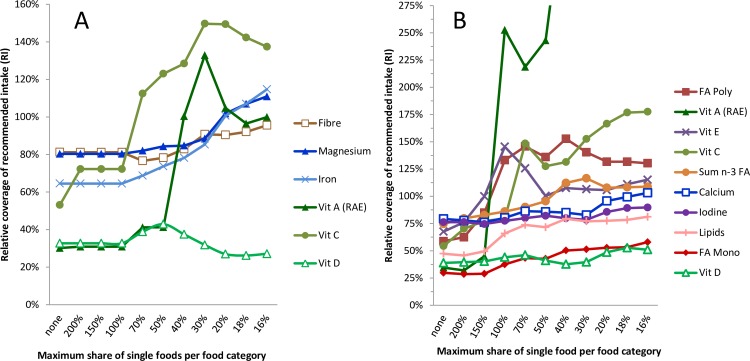
**Effects of diversification through lowering the maximum allowed relative share of a single food within each food category on the contents of nutrients that were below 95% of the RI in the non-diversified form (Table 3) A) Food basket CA B) Food basket DG.** Abbreviations: Vit: vitamin; RAE: retinol equivalent units; n-3 FA: omega-3 fatty acids; FA mono: monounsaturated fatty acids; FA poly: polyunsaturated fatty acids.

If only cost and cultural acceptability (CA) were considered, the one-day FB was 50–90% deficient in fibre, magnesium, iron, and vitamin C and more than 50% deficient in vitamins A and D (Fig 3A). Similarly, if DG alone was considered, the one-day FB was 50–90% deficient in polyunsaturated and omega-3 FA, vitamins C and E, calcium, iodine, and >50% deficient in vitamins A and D, total lipids, and monounsaturated FA (Fig 3B). The amount of nutrients, with the exception of vitamin D and monounsaturated FA, in CA and DG increased above 100% of the recommendations after increasing the variety of foods in these FBs (Fig 3).

**Fig 4:**
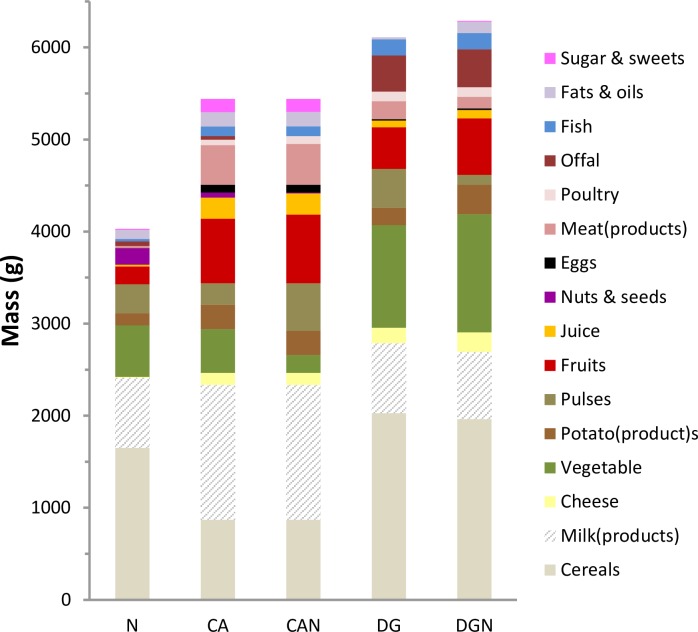
Composition of the diversified FBs by food categories. To make the effects of the different sets of constraints more evident, the category “Meat & meat products” used in the Danish consumption survey [26] was split up into “Meat(products)” and “Offal”; pulses are indicated as a separate category (part of the “Vegetable” group in the consumption survey; and “Nuts & seeds” are indicated separately from the “Fruit” group.

Although being isocaloric, the diversified N had a considerably lower weight (~4.0 kg) than the other FBs (~5.4–6.3 kg). Implementation of constraints on cultural acceptability (CA) and health promotion (DG) had a stronger influence on the composition of the combined FBs (CAN and DGN) than constraints on nutritional adequacy (N) (Fig 4). Compared to N, CA and CAN contained more milk(products), fruits, and meat and DG and DGN contained more cereals, vegetable, fruits and offal (Fig 4).

**Fig 5:**
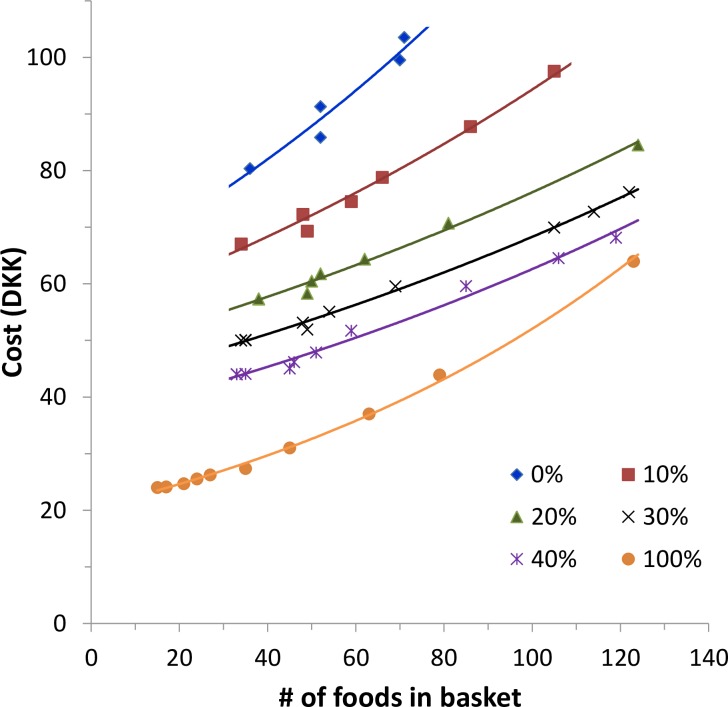
Dependence of price and food variety after step-wise release of the cultural acceptability constraint in the CAN. Percentages indicate the tolerated deviation from the averagely consumed weight of food categories by the Danish population [26]. The initial CAN has 0% allowed deviation (top line).

Already a moderate release of the cultural acceptability constraint MRD (10% deviation from the averagely consumed weight of each food category allowed) resulted in a significant drop in the minimal cost and increased food variety of CAN (Fig 5). After increasing the MRD to 20%, food variety did not increase further and additional increase of MRD resulted in reduced cost only. Allowing a 40% MRD resulted in a food variety that was comparable to that of N and cost only ~DKK 1 (€ 0.13) more than N.

## Discussion

The present study shows that a systematically structured approach using LP to increase the variety of foods illustrates how culturally acceptable FBs can be constructed for the lowest possible cost.

Similar to earlier investigations it was found that very few foods are needed to meet both nutrient and dietary recommendations for the lowest cost [19]. The Danish FBDGs, when applied as constraints during the construction of FBs with increased food variety, appear to result in coverage of all recommended intakes [6] except vitamin D and monounsaturated FA (Fig 3). One of the advantages of using a methodology such as LP is that it provides a systematic approach to confirm e.g. in dietary guidelines whether or not nutritionally adequacy is assured in different contexts for different populations.

This study illustrates how a low cost diet can be designed to be both nutritionally adequate and to prevent NCDs in the Danish population. The cost was primarily determined by key micronutrients: vitamins D, C, B2 and iodine, potassium, and calcium. When these key nutrients were incorporated at the recommended levels all other macro- and micro-nutrients were automatically present in sufficient amounts. The lowest cost was best achieved by including the foods that contain high levels of these key nutrients: whole-grain products, root vegetables, fatty fish and milk. The important role of foods rich in vitamin D was also recognised by Swedish [30] and Slovenian investigators (Gregoric et al., unpublished report for the Ministry of Labour, Family and Social Affairs of Slovenia, 2009). It may be difficult to cover vitamin D recommendations from foods alone [38] and exposure to sunlight (UV-B) is also recommended [39]. Alternatively, vitamin D supplements or vitamin D fortification [40] may be recommended especially during the winter and early spring in the Northern hemisphere [41].

One of the strengths of the LP-model is that it can facilitate the generation of a household food basket for the period of one month, or longer or shorter, at the lowest possible cost and enables recipe development for complete meals (Table 4). The minimum cost of the one-month household FB of type N for a Danish family of four was ~DKK 54 (~€ 7.2) per day. This cost is similar to that found by French investigators (€ 3.20 and € 3.40 per day for a woman and man, respectively) [42] but less than half that found in the United States ($ 18.60, ~€ 17, for a family of four) [43]. In 2007, Danish investigators estimated that a Danish family, following the official Danish FBDGs, would have to spend on average DKK 171 (~€ 23) per day [44], corresponding to between 3-fold the cost of N and about 40% more than the cost of CAN, based on average Danish food prices. In 2010, Danish statisticians reported that the average Danish household (2 adults and 1.8 children) actually spent DKK 140 (~€ 19) on their household food budget, excluding foods bought outside the home [45]. Lower socioeconomic households spend around 20–25% less than the higher socioeconomic households (standardized for household size and composition). However, based on the average disposable income for two Danish adults on average salaries and using the DGN model, between 6% and 11% of their household income is needed to cover its cost. In contrast, families suffering from unemployment or dependant on benefits would have to spend between 10% and 18% of their income [46]. The percentage of disposable income needed in Ireland to cover the cost of a healthy diet was estimated to be up to 69% of income [47], between 30% and 48% in Australia [48,49] and 30% in Canada [50]. In addition to the differences in national food prices, this wide range in % income probably arises from the different methods available to calculate the cost of a healthy diet. One advantage of the LP method is that so-called “unhealthy” foods (i.e. those with unfavourable nutrient profiles) can be combined with “healthy” foods to design an overall healthy diet [51].

Most consumers are unlikely to adopt food basket recommendations unless they consider these practical, feasible, culturally acceptable, familiar and sufficiently varied in number [42]. Consumers in France eat on average around 50 different foods per week [52] compared with the household food basket for a month in this study, which contains twice that number (Table 4). Eating patterns are notoriously difficult to change. There are many barriers e.g. cost, taste, habit and others to changing to a healthy diet [53] especially in low socioeconomic groups. For example in Denmark, consumers prefer meat, meat products, eggs, sweets, sugar-sweetened soft drinks, and alcoholic drinks [26]. However when these foods were selected by the linear programming method, e.g. because of their cultural acceptability only, the overall cost increased and it was not possible to meet the nutrient and dietary recommendations. Indeed the gap between a culturally acceptable diet and the Nordic nutrition recommendations is wide. Almost no meat, juice, and sweets and considerably less milk products were in the LP designed food baskets of types DG and DGN. In contrast, they contained more than twice as much cereals and vegetables and more liver compared with the CA. The required change seems quite dramatic and so households have to be quite determined and resourceful to incorporate these newly designed FBs into their daily routine and to compile this big change in food variety into new recipes and meals that their families will eat. Cultural acceptability, however, can only be achieved at the expense of cost (Fig 5) and in some cases inability to meet nutrient recommendations.

The cost data presented here are based on the purchase price alone. However foods once purchased have to be converted into appetising meals and so additional resources are needed for: transport; equipment; storage, preparation and cooking facilities; utensils to serve meals; energy for hot water, food storage and preparation (refrigerating, freezing, cooking); time to prepare meals (and assuming person preparing food might otherwise be earning) [54,55]; drinks, spices, and unavoidable food waste. Food prices vary due to different national retail policies, marketing practices, fixed retail packaging sizes, seasonal local availability and price fluctuations, and volatility on the global market [56]. There are also hidden costs related to how households can plan and cook new recipes and prepare meals. Similarly, households´ nutritional needs vary depending on its number of inhabitants, their age and level of physical activity and whether or not they suffer from overweight. Therefore food baskets must be calculated at a national or sub-national level to consider the local context and costs. Indeed a reliable, sufficiently detailed, international database on food prices [57] would facilitate the possibility of authorities being able to design relevant food baskets and dietary guidelines using the LP methodology.

## Conclusion

Nutritional adequacy, health-promoting, NCD-preventing properties, and cultural acceptability are all constraints that need to be addressed and LP is a method that can help solve this complex task. When designing low cost national food baskets their feasibility and implementation has to be investigated via intervention studies. Feasible food baskets, which are readily accepted by low socioeconomic groups, could serve as the basis for national food based dietary guidelines that can help reduce diet-related health inequalities. National and international authorities could, by using linear programming methods, design dietary guidelines that are more cost-effective in preventing micronutrient deficiencies and diet-related NCDs.

## Supporting Information

S1 Table Food Prices in the Larger Copenhagen Area. The file provides the food category, the name of the food item, and the corresponding average, maximum, and minimum price.
(CSV)
